# Rapunzel Syndrome: A Case of Trichobezoar with Small Bowel Complications

**DOI:** 10.1055/s-0042-1757777

**Published:** 2022-11-06

**Authors:** Ramakrishna Narra, Anusha Guntamukkala, Chanda Bhaskara Rao, Tanveer Begum

**Affiliations:** 1 Department of Radio-diagnosis, Katuri Medical College, Guntur, Andhra Pradesh, India; 2 Department of Pediatric Surgery, Guntur Medical College, Guntur, Andhra Pradesh, India; 3 Department of Pediatrics, Katuri Medical College, Guntur, Andhra Pradesh, India

**Keywords:** trichobezoar, Rapunzel syndrome, intussusceptions, contrast-enhanced computed tomography

## Abstract

**Introduction** Rapunzel syndrome is characterized by a large trichobezoar in the stomach with a tail extending beyond the pylorus into the small bowel, causing mechanical obstruction of the small bowel. A 7-year-old girl presented to the emergency room with severe epigastric pain. Computed tomography suggested trichobezoar causing jejuno-jejunal intussusceptions, bowel wall thickening, and dilated small bowel loops proximal to the obstruction. On laparotomy, two concealed perforations were noted at the duodenojejunal (DJ) junction and 40 cm distal to the DJ junction. An enterotomy incision was given at the antimesenteric border of the distal jejunal perforation site, and the mass was successfully extracted. Primary repair was done at the DJ perforation site, and resection was followed by an end-to-end anastomosis at the distal jejunal perforation site. Surgery confirmed a complex mass of tangled hair within the gastric cavity with a tail extending into the pylorus of the stomach and small intestine, consistent with trichobezoar.

**Conclusion** Computed tomography is superior to other radiological imaging modalities for diagnosing trichobezoars as it helps diagnose and demonstrate mechanical bowel complications.

Bezoars are formed by indigestible material within the gastrointestinal tract that eventually transforms into an agglomerate nondigestible mass. They are classified according to their contents as phytobezoars (composed of fruit fibers or plants), lactobezoars (composed of milk), trichobezoars (concretions of hair), and pharmacobezoars (composed of medications). 1 Trichobezoars are associated with psychiatric illnesses such as trichotillomania and trichophagia. 2 Approximately 1% of patients suffering from trichophagia develop trichobezoar. 3 Rapunzel syndrome is characterized by the presence of a trichobezoar in the stomach with a tail that extends beyond the pylorus and causes mechanical bowel obstruction. 4 We here report a case of Rapunzel syndrome with small bowel complications.

## Case Report

A 7-year-old female child presented to the emergency room with severe colicky epigastric pain and nonbilious vomiting for 5 days. The patient had a past medical history of trichotillomania and trichophagia. No signs of mental retardation. On general examination, the patient appeared pale. She was afebrile, and all other vital signs were normal for her age (heart rate: 115 beats/min, respiratory rate: 18 breaths/min, blood pressure: 110/70 mm Hg, oxygen saturations: 99% on room air, oxygen saturations: 100% on room air, and capillary refill: <2 seconds). On abdominal examination, a firm nontender mass was palpated, extending from epigastrium to left hypochondrium. Approximately 7.2 × 5.5 cm ^2^ firm, nontender, well-delineated lump occupying the epigastrium with a smooth surface was noted. There was no rigidity or guarding. Routine blood and urine investigations performed demonstrated mild anemia. The rest of the parameters were within normal limits (hemoglobin: 9 g%, total leukocyte count: 9500/cumm, differential leucocyte count neutrophils: 75%, lymphocytes: 22%, eosinophils: 02%, macrophages: 01%, red blood cell: hypochromic and microcytic, platelets: 2.5 lakh/cumm). Urine examination, liver function tests, and serum electrolytes were within normal limits. Blood urea 22 mg% and serum creatinine 0.8% mg%. Total serum proteins 3.5 g%, albumin 1.2 g%, globulin 2.3 g%, blood glucose 105 mg%, iron 23 microgm%, ferritin 11 microgm%, and total iron binding capacity raised. She appeared well nourished and weighed 24.1 kg. Her height is 121 cm

**Fig. 1:**
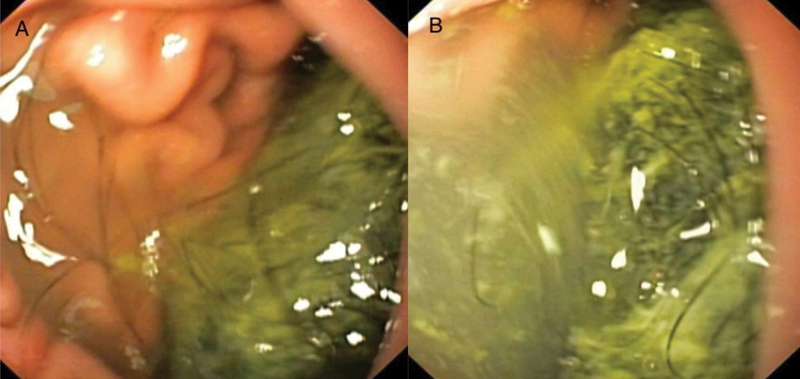
( **A, B** ) Upper gastrointestinal endoscopy images demonstrating large trichobezoar in the body of the stomach extending into the antrum and pylorus obstructing the further passage of endoscope.

**Fig. 2:**
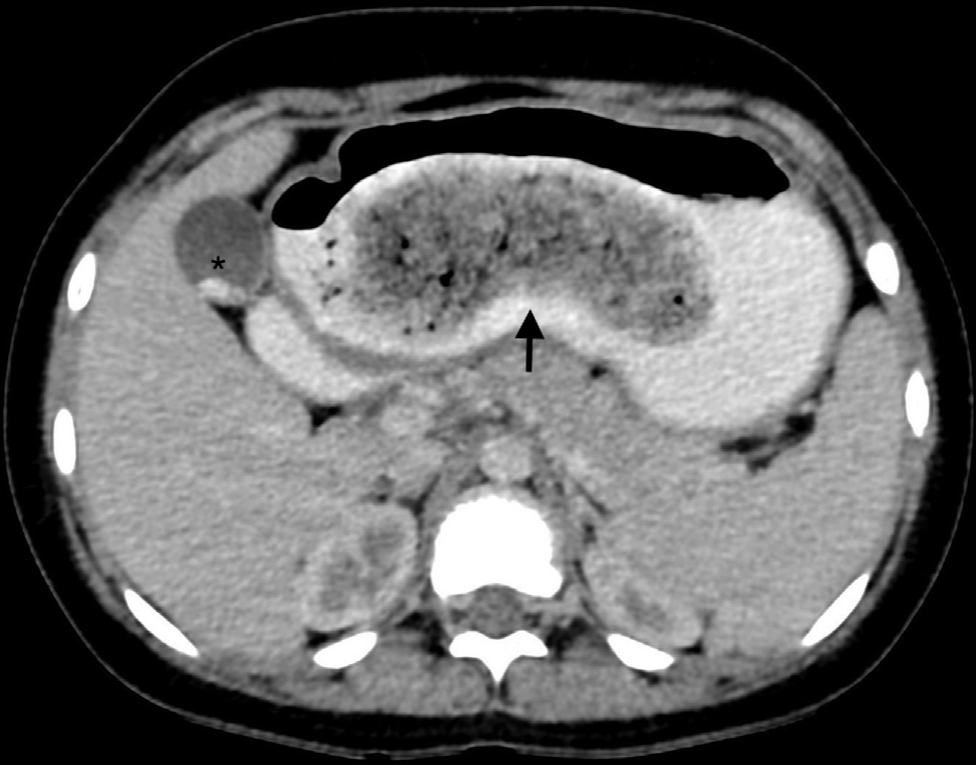
Axial oral and intravenous contrast-enhanced computed tomographic scan of abdomen demonstrating a heterogenous density lesion (black arrow) within the lumen of stomach with “mottled” appearance and clearly outlined by intraluminal oral contrast coincidental gall bladder calculi (asterisk).

The plain radiograph demonstrated a distended stomach gas shadow. Routine ultrasound abdomen showed nonspecific echogenicity with intense acoustic shadow within the stomach and pylorus region. Upper gastrointestinal endoscopy revealed a large trichobezoar ( Fig. 1A, B ) filling the gastric lumen and obscuring the distal end of the stomach. Contrast-enhanced computed tomographic scan of the abdomen and pelvis performed demonstrated a nonenhancing, well-circumscribed heterogeneous filling defect with a typical “mottled gas pattern” due to entrapped air in the body and pylorus of the stomach and tail extending into the duodenum, jejunum, and proximal part of the ileum without any attachment to the bowel wall with thickening of the wall of the duodenum ( Figs. 2 3 4 ). In the proximal jejunum, telescoping of the small bowel loops was observed, suggestive of jejuno-jejunal intussusceptions ( Fig. 5 ). In addition, we noted dilated small bowel loops proximal to the obstruction and bowel wall thickening. Findings were consistent with a large trichobezoar causing jejuno-jejunal intussusceptions. Due to its large size, endoscopic removal was not feasible, and exploratory laparotomy was performed under general anesthesia.

**Fig. 3:**
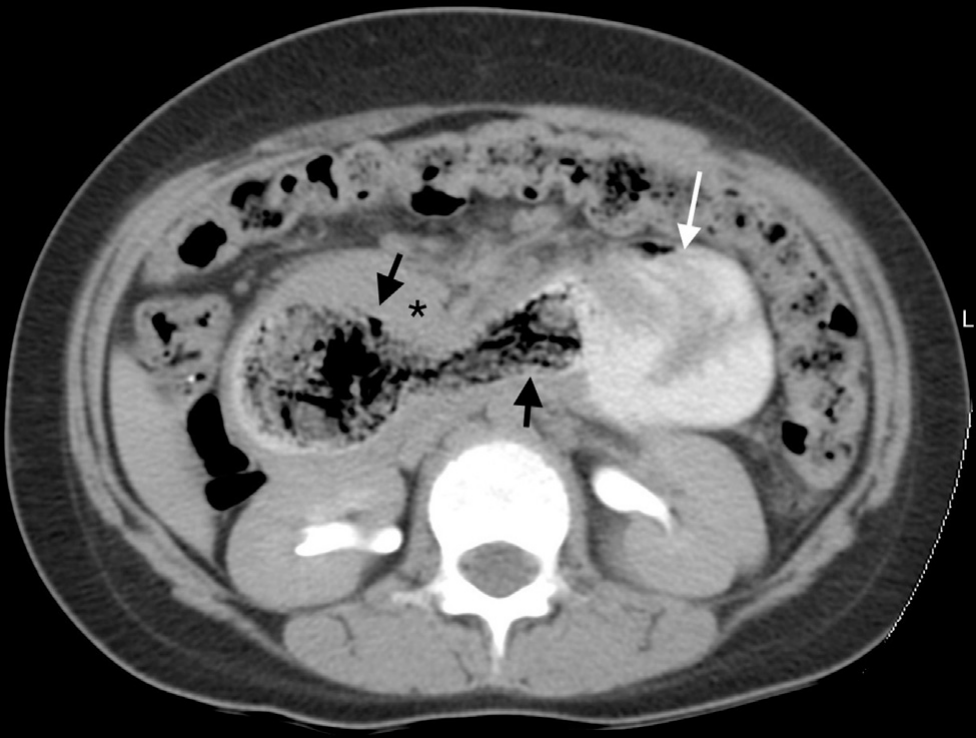
Axial oral and intravenous contrast-enhanced computed tomographic scan of abdomen demonstrating a heterogenous density lesion (black arrows) within the lumen of duodenum with “mottled” appearance and clearly outlined by intraluminal oral contrast. Note the dilated proximal jejunal loop (white arrow) secondary to the distal jejuno-jejunal intussusceptions and duodenal wall thickening (black asterisk).

**Fig. 4:**
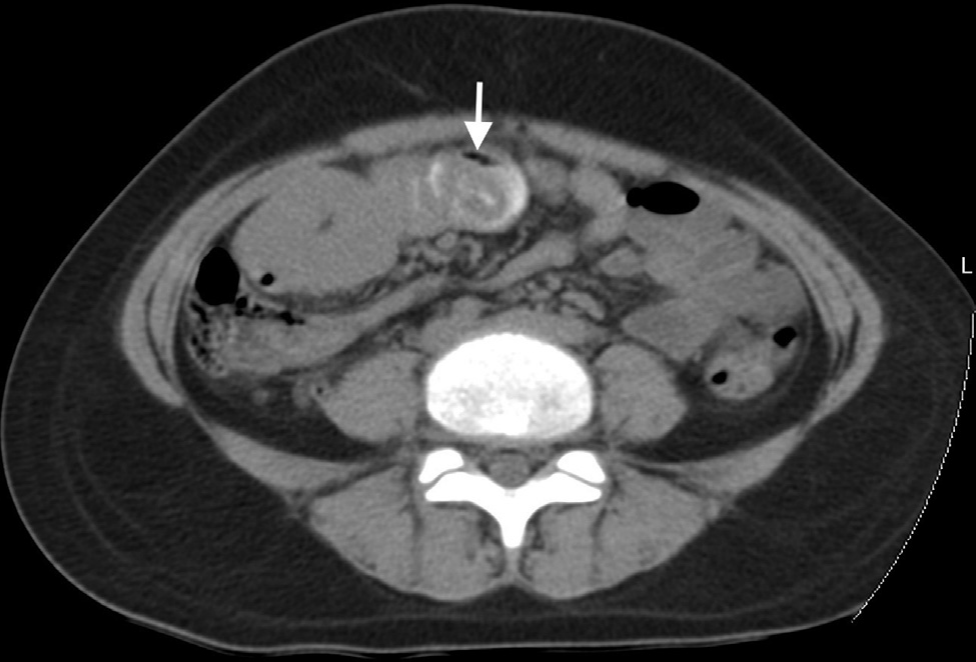
Axial oral contrast-enhanced computed tomographic scan of abdomen demonstrating the telescoping of the jejunal loops suggestive of jejuno-jejunal intussusception (white arrow) with thickening of adjacent jejunal loops.

**Fig. 5:**
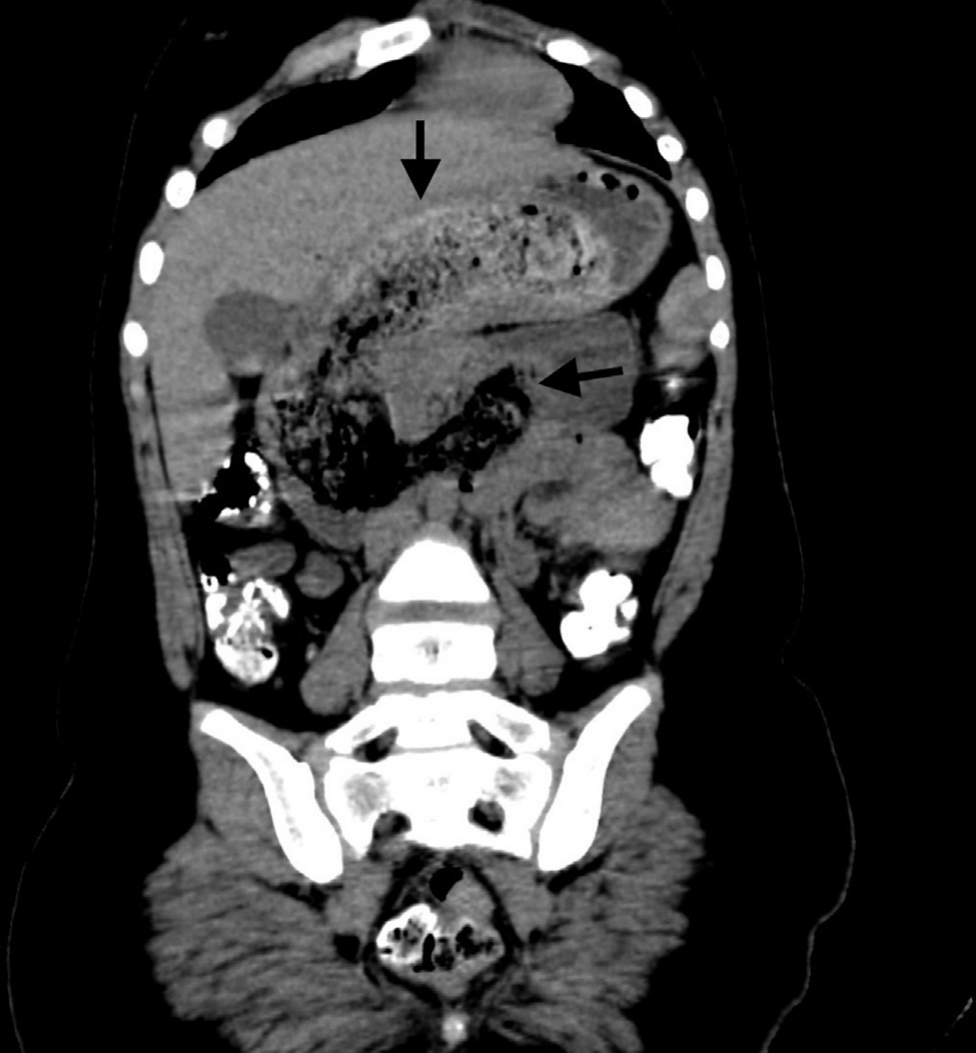
Coronal multiplanar reformation of oral and contrast-enhanced computed tomographic of abdomen demonstrating the trichobezoar (black arrows) outlined by oral contrast media and contents in the stomach, duodenum, and proximal jejunum.

**Fig. 6:**
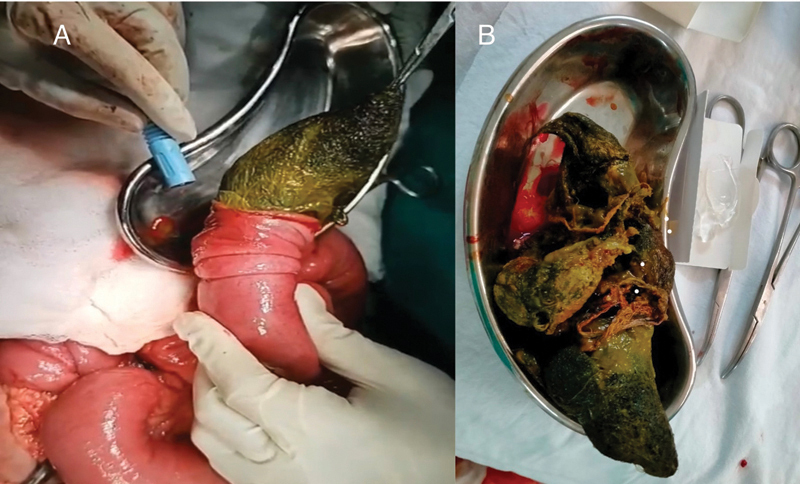
Operative ( **A** ) and postextraction ( **B** ) images demonstrating the trichobezoar extraction by laparotomy and enterotomy, and postoperative specimen.

A midline laparotomy incision was given, and a duodenojejunal (DJ) junction was noted to the left of the L1 vertebra. Masses were palpated at the stomach and small bowel. Two concealed perforations were pointed out at the DJ junction and 40 cm distal to the DJ junction. Trichobezoar was successfully removed through a separate enterotomy incision at the antimesenteric border of the distal jejunal perforation segment as the perforation site is close to mesentery ( Fig. 6 ). DJ junction perforation was closed using 3–0 silk in two layers. Resection and end-to-end anastomosis were performed at the distal jejunal perforation site. Given trichotillomania, the patient was further referred for psychiatric evaluation. Postsurgical follow-up was uneventful, and no significant complications occurred.

## Discussion

Rapunzel was a fairy tale character with long hair. Because of the resemblance of the tail of a trichobezoar extending into the small intestine to the hair of Rapunzel, this condition was named Rapunzel syndrome. 5 There have been only 45 cases of Rapunzel syndrome reported, and less than 15 cases were reported with small bowel complications. 6

Trichobezoar is a complex mass made of swallowed hair and makes up less than 6% of all bezoars. 7 Human hair is resistant to digestion, and the ingested hair pieces are mixed with mucus and food particles over a long period, forming a thin encapsulated mass. In young females, trichobezoars are often associated with psychiatric illnesses such as trichotillomania (hair-pulling) and trichophagia (hair swallowing). 8 Trichotillomania involves pulling the hair to the point of alopecia and is mainly performed on the scalp, although eyelashes, eyebrows, and the axilla are all susceptible. Only 30% of these patients will also engage in trichophagia, and of those that do, only 1% will eventually develop a trichobezoar that requires surgical extraction. 3 Patients with trichobezoar usually present with nausea, vomiting, abdomen pain, gastric ulcers, hematemesis, perforation, and intestinal obstruction. 9 Small bowel intussusception with or without small bowel obstruction(transient) may be an associated complication. Regardless of the cause, it is important to remember, particularly in recurrent cases, that bezoars may seldom be the undeclared cause of intussusception. In some cases, the intussusceptions may be at multiple sites and are transient. 10

Nour et al reported a rare case of Rapunzel syndrome with generalized edema. Protein-losing enteropathy, poor intake, malabsorption, and bacterial overgrowth may contribute to hypoalbuminemia that occurs insidiously over a long period. 11

The abdominal radiograph is nonspecific and may demonstrate a distended stomach shadow with an intragastric mottled gas pattern outlined by fundal gas, which may resemble a food-filled stomach. Barium studies may show an intraluminal filling defect with a mottled gas pattern without attachment to the bowel wall. Transabdominal ultrasound may demonstrate a hyperechoic curvilinear mass associated with posterior acoustic shadowing within the stomach and pylorus region. 12 Computed tomography is a better radiological modality for demonstrating the size and configuration of the bezoar. It shows the entire length of the bezoar as an intragastric well-circumscribed mass consisting of a “mottled gas pattern” or “compressed concentric rings” pattern due to the presence of entrapped air and food debris and tail extending up to the duodenum or jejunum with oral contrast material dispersed within the mass and surrounding it. Contrast-enhanced computed tomography scans can differentiate the bezoar from the gastrointestinal tumors such as a gastrointestinal stromal tumor extending into the stomach lumen and other bezoars like phytobezoar. Oral contrast can trace a normal stomach wall separately from the lesion. 13 We can notice mucosal edema and wall thickening in the duodenum and jejunum. Oral contrast demonstrates intussusceptions. 14

With its limitations, magnetic resonance imaging is less beneficial than computed tomography for diagnosing trichobezoar. The upper gastrointestinal endoscopy may demonstrate a complex mass of hair within the stomach and detect other complications such as gastric inflammation and ulcers.

Enzymatic degradation and medical treatment of trichobezoars are futile as they resist them. Endoscopic removal of trichobezoars is mostly ineffective. The large size and dense composition of the bezoar limit its endoscopic fragmentation. On endoscopic removal, we should consider the possibility of migration of lysed bezoar fragments into the small bowel, causing a secondary obstruction. Surgical removal through laparotomy is the treatment of choice for trichobezoars given its high success rate, low complication rate, low complexity, and potential to examine the small bowel and management of intussusception. Long-term psychiatric counselling is essential to reduce the risk of recurrence of trichotillomania and trichophagia. 15

## Conclusions

We present a case of Rapunzel syndrome with small bowel complications. In young females with underlying psychiatric illness presenting with the features of small bowel obstruction, one should exclude the possibility of trichobezoar. Computed tomography is superior to other radiological imaging modalities for diagnosing trichobezoars as it helps diagnose and demonstrate mechanical bowel complications.
